# Metabolomics of Red Wines Aged Traditionally, with Chips or Staves

**DOI:** 10.3390/foods13020196

**Published:** 2024-01-07

**Authors:** Georgiana-Diana Dumitriu (Gabur), Fernando Sánchez-Suárez, Rafael A. Peinado, Valeriu V. Cotea, Nieves López de Lerma, Iulian Gabur, Violeta Simioniuc

**Affiliations:** 1 Faculty of Horticulture, Iasi University of Life Sciences, 700490 Iasi, Romania; diana.gabur@uaiasi.ro (G.-D.D.); vcotea@uaiasi.ro (V.V.C.); 2 Agricultural Chemistry, Soil Science and Microbiology Department, University of Córdoba, Campus of Rabanales, N-IV Road, Km 396, 14071 Córdoba, Spain; g62sasuf@uco.es (F.S.-S.); b92lolem@uco.es (N.L.d.L.); 3 Department of Plant Science, Iasi University of Life Sciences, 700490 Iasi, Romania; gaburi@uaiasi.ro

**Keywords:** ageing system, odour activity values, oak chips, staves, barrels, toast degree

## Abstract

Traditionally and alternatively aged wines’ odour activity values (OAVs) are investigated to differentiate and highlight the differences between the selected methods. An analysis of the volatile aroma compounds of wines derived from ageing in barrels, oak chips, and staves was performed using stir bar sorptive extraction chromatography–mass spectroscopy (SBSE-GC-MS). The results showed that alcohols, esters, and oak compounds were the main contributors to aroma, and their OAVs were higher in the stave samples after 3 months than in the samples from the other two systems of ageing. Furthermore, wines aged with staves have stronger fruity, spiced, and woody aromas, while samples aged in barrels present more chemistry-driven, floral, caramelly, and creamy aromas. The staves—medium plus toast (SMPT at 3 months > 225) and chips—medium plus toast (CMPT at 3 months > 170) showed the highest levels of aromatic series, suggesting that alternative systems provided more powerful aromas than traditional systems, such as barrels—medium plus toast (BMPT at 3 months > 150). A principal component analysis (PCA), orthogonal partial least squares (OPLS) analysis, and cluster analysis allowed for a clear differentiation to be made between red wines according to ageing systems and ageing times. The odour activity values fingerprint in winemaking is a feasible approach to characterise and distinguish wines. Moreover, OAVs provide important information on the effects of production methods on wine quality and aroma profile.

## 1. Introduction

Wood ageing is a crucial step in the production of red wines, as this stage generates unique sensory characteristics for the final product that would not be apparent when using other conservation containers, such as stainless steel tanks or amphoras. Traditionally, wines are aged in wooden barrels [1,2]. During the ageing process, many chemical reactions take place between wines and barrel wood. For example, oxygen comes into contact with wine through the wooden container, which is a process that plays a central role and is fundamental to the red wine aroma profile [3]. A pivotal sensory attribute establishing the quality of wine is given by the volatile compound profile present in the samples. During wine ageing, the generation, transformation, and degradation processes of volatile compounds are complicated, inconsistent, and instable [4]. Comprehending the evolution of these aroma profiles in wines depends on the ability to monitor them correctly during the ageing process.

Barrels used in winemaking come with a series of limits due to time, costs, required space, evaporation loss, and reduced extractable compounds [5]. To overcome most of these drawbacks, research on alternative ageing methods is required. Alternative ageing methods with various small pieces of oak have become increasingly utilised in the past few years due to their low costs, ease of use, and the accumulation of age-specific aromas in a short time period. Moreover, these alternative methods are eco-friendly, reduce environmental impacts, and limit climate change by using only small fragments of wood, thus favouring the creation of sustainable winemaking practices. García-Alcaraz et al. [6] compared three alternative ageing processes with the use of a new oak barrel and concluded that the use of oak chips for winemaking reduced costs by 200 times and produced similar quality profiles. Moreover, alternative methods are needed, as chips can have a quarter of the environmental impact compared with traditional barrel ageing. This is similar to oak staves, which have a 17–20% lower cost and a 34% lower environmental impact compared to barrels.

The chemical composition and organoleptic characteristics of wines are influenced by a great number of factors linked to the ageing process. These factors are related to the nature of wood (origin and type of wood); the characteristics of the process of making the oak chips, staves, and barrels (intensity of the toasting process, size, and quantity of pieces); or the ageing process itself (ageing time) [7,8,9].

During ageing in barrels, wines are subjected to a more oxidative environment than wines aged with staves or chips due to the greater extent of oxygen that enters through the barrel walls. This process affects the hydrolysis of aroma precursors and the formation of reaction products resulting from the interaction of volatile and non-volatile compounds in oak and wine. Oak-derived volatile compounds are responsible for most of the sensory effects of red wines aged in oak barrels [10]. Oak-derived volatile compounds include lactones and their two isomers (*cis*- and *trans*), furanones, volatile phenols, volatile aldehydes, esters, norisoprenoids, pyranones, and acetic acid, among others.

Regardless of the wine ageing system, namely traditional or alternative, wood compounds have to undergo a toasting process. Wood toasting can be realised by fire, gas, steam, or boiling water, and it is a process that influences the chemical and physical structure of wood. Four types of toasting are commonly used in winemaking: light, medium, medium plus, and heavy. Light toasting is carried out at temperatures between 120 and 180 °C, while medium and medium plus toast use temperatures of >190 °C and ≥250 °C, and heavy toasting is carried out between 250 and 280 °C [11]. Heavy-toasted chips release fewer oak lactones into wines than light-toasted chips due to the thermochemical degradation of these heat-sensitive compounds or their loss by volatilisation when the oak wood is subjected to very high temperatures [12]. The toasting process increases the number of volatile compounds and leads to the development of new compounds. In the literature, higher toasting temperatures have been linked to an enhanced perception of fruity aromas in finished wines [13]. Heavy toasting was found to have a low potential for extracting volatile compounds, while [14] medium toasting coincides with the maximum synthesis of these volatile compounds [15]. Furanic aldehydes are formed by the thermal degradation of celluloses and hemicelluloses. Furfural and 5-methylfurfural are degradation products of hexoses and pentoses, reminiscent of roast aromas and almond flavours. The thermal degradation of lignin leads to the formation of methoxylated volatile phenols and phenolic aldehydes associated with spice, smoke, and vanilla aromas. *Cis*- and *trans*-β-methyl-γ-octalactone, two diastereomers of oak lactone, arise from the thermal degradation of woody substances, which contribute to the coconut and wood aromas in wine. Thus, it is clear that the variety of volatile compounds derived from oak wood can contribute significantly to the richness and flavour complexity of wines.

High-quality red wines are generally those that spend an important period in wood barrels and are subjected to a long ageing process. Red wine aroma profiles are the result of a close relationship between ageing time and wine aroma compounds. The relationship between the concentration and the maximum perception odour threshold, referred to as the “odour activity value” (OAV), must be considered as the sole principle for estimating the contribution of each compound to aroma. However, antagonistic and additive effects between different aroma components can occur in wines [16]. Moreover, since a single compound usually exhibits multiple aromas, it is difficult to establish or evaluate global aroma profiles based only on the OAVs of the volatiles. Therefore, the OAVs of aroma compounds with similar descriptors have to be categorised into aromatic series. In the case of wine, which has a complex chemical composition, fingerprinting with omics methods has been used for discrimination, identification, quality control, and to study the effect of wine volatiles on the overall wine aroma profile [17].

In this study, stir bar sorptive extraction chromatography–mass spectroscopy (SBSE-GC-MS) was used to detect aroma compounds, which primarily contribute to the changes in metabolite profiles during red wine ageing. Improving wine aroma profiles depends on the winemaker’s capacity to optimise alternative ageing systems. Therefore, this study compares alternative (oak chips and staves) and traditional (barrel) winemaking processes and explores the possibility of improving the aroma quality of red wines. Moreover, monitoring volatile compound transformation during ageing processes helps with deciphering the enological characteristics of ageing systems (traditional and alternative). The results will provide winemakers with sustainable methods to optimise wine quality by improving the ageing process.

## 2. Materials and Methods

### 2.1. Chemical Standards

The identification and quantification of aroma compounds were carried out with standard solutions of pure compounds of analytical grade, purchased from Sigma-Aldrich (St. Louis, MO, USA), Merck (Rahway, NJ, USA), and Fluka (Buchs, Switzerland). Pure water was obtained from a Milli-Q purification system (Millipore, Burlington, MA, USA).

### 2.2. Ageing Conditions

Experiments were carried out using grapes of the variety Fetească neagră (*V. vinifera*) on a pilot winemaking scale. The chemical parameters of the control wines were pH 3.68, titratable acidity (g tartaric acid/L) 5.80, volatile acidity (g acetic acid/L) 0.48, and alcoholic strength (% *v*/*v*) 13.9. Samples obtained using the common red wine processing method were divided as follows: (a) 5 g/L of oak chips (C) (0.5 cm × 1.5 cm × 0.2 cm) was added to 250 L of wine in stainless steel tanks; (b) 4 pieces/L of staves (S) (1 cm × 10 cm × 1 cm) was also added to 250 L of wine in stainless steel tanks; and (c) new barrels (B) of 225 L were used for ageing wines. Samples were coded as chips—C; staves—S; and barrels—B. Two toasting degrees were used: medium toast (MT) and medium plus toast (MPT). Two aging periods were used: 1.5 and 3 months. Every variant was performed in triplicate. *Quercus petraea* oak species was used for the alternative ageing systems, and the traditional system used *Quercus robur* oak species. The barrels were manufactured by Unicom Wood Production, and oak chips and staves were supplied by Amedee. After ageing, wines were then bottled and stored at 12 °C.

### 2.3. Identification and Quantification of Aroma Compounds

#### 2.3.1. Major Volatiles

Gas chromatograph and a flame ionisation detector (GC-FID) was employed for the analysis of major volatile compounds present in 10 mg/L samples. Samples were directly injected using an HP 6890 gas chromatograph (GC) and a flame ionisation detector (FID), along with a capillary column, CP-WAX 57 CB (50 m in length, 0.25 mm in internal diameter, and 0.4 μm in coating thickness). Quantitative data were obtained by using the calibration curve for each compound [18,19]. We utilised a split ratio of 30:1, an FID, and a temperature program that consisted of an initial temperature of 50 °C (15 min), a 4 °C min^−1^ ramp, and a final temperature of 190 °C (35 min). The injector and detector temperatures were 270 and 300 °C, respectively. The flow rate of carrier gas (helium) was initially set at 0.7 mL min^−1^ (16 min) and then increased by a 0.2 mL min^−1^ ramp to the final value (1.1 mL min^−1^) for 52 min. An amount of 0.5 μL aliquots of 10 mL wine samples previously supplied with 1 mL of 4-methyl-2-pentanol as an internal standard (1 g/L) were injected into the split/splitless injector of the GC instrument. Tartaric acid in the wine was removed by precipitation with 0.2 g of calcium carbonate, followed by centrifugation at 300 g and 4 °C.

#### 2.3.2. Minor Volatiles

##### Extraction of Minor Aroma

SBSE was used for the extraction of minor aroma compounds, according to Lopez de Lerma et al. [20]. Each wine sample was diluted in a proportion of 1:10 with a hydro-ethanolic solution containing 12% ethanol (*v*/*v*), and which was previously adjusted to pH 3.5 with 2.6 g/L tartaric acid and 2.2 g/L potassium bitartrate. A stir bar (0.5 mm film thickness, 10 mm length, Gerstel GmbH, Mulheim an der Ruhr, Germany) coated with PDMS was placed in a 10 mL glass headspace vial containing 10 mL of the diluted sample and 0.1 mL of a solution of ethyl nonanoate (0.45 mg/L) as an internal standard. The vial was sealed with a Teflon-coated crimp cap. The stir bar was stirred at 1500 rpm for 100 min at 25 °C, and the stir bar was transferred into a glass thermal desorption tube for GC–MS analysis.

##### Determination of Minor Aroma

The glass thermodesorption tube was inserted into the Gerstel TDS 2 thermodesorption system connected to the GC-MS model. The stir bar was heated to release and transfer the extracts into a cooled injection system/programmed temperature evaporator (CIS 4 PTV) with a Tenax adsorption tube. Thermal desorption was performed with a temperature program of 35 °C, ramping from 120 °C min^−1^ to 280 °C and held for 10 min; the helium flow rate was 3 mL/min. The CIS injector was maintained at 25 °C for the entire desorption time and then ramped to 280 °C with a ramp of 12 °C s^−1^ in split-less mode and held for 7 min.

The GC was equipped with an Agilent-19091S capillary column (30 m × 0.25 mm i.d., 0.25 μm film thickness). Helium was used as the carrier gas with a column flow rate of 1 mL min^−1^. The temperature of the GC oven was fixed as follows: 50 °C for 2 min, ramped at 4 °C min^−1^ to 190 °C, and held for 10 min. The mass detector was used in scan mode, and the mass range investigated included values from 39 to 300 *m*/*z*.

To identify and confirm volatile compounds, retention times, spectral libraries provided by Wiley (version 7 N), and pure chemical compounds were used. Each compound was quantified from its calibration curve, which was generated by using standard solutions of known concentrations previously subjected to the same treatment as the samples in conjunction with the target and by qualifying values selected for each compound using Hewlett-Packard Chemstation (Palo Alto, CA, USA).

### 2.4. Odour Activity Values (OAVs)

Odour activity value (*OAV*) was determined to assess the contribution of a chemical compound to the aroma of a wine sample. The *OAV* is a measure of the importance of a particular compound to the odour of a sample. It was calculated as the ratio between the concentration of a single compound and the perception threshold found in the literature. For all of the compounds obtained from the wine samples, the *OAV* was calculated as the ratio of the concentration of each compound to the respective odour threshold using Equation (1):

(1) $$OAV=\frac{C}{T}$$

where *C* is the concentration of the individual volatile compound in the wine sample, and *T* is the odour threshold of this compound, which was obtained from data reported in the literature.

### 2.5. Aroma Series

The aroma series is described as a group of volatile compounds with similar aroma descriptions, and its value is given by the sum of the OAVs of the compounds that make up the series. The same compound may be included in one or more aromatic series in accordance with its aromatic descriptors. Eleven aroma series were obtained, namely chemistry, floral, green, caramelly, fruity, creamy, citrus, honey, waxy, spice, and woody. In general, an odour-active compound with an *OAV* ≥ 1 is expected to potentially contribute to the overall odour impression of a sample [21].

### 2.6. Statistical Treatment

To identify statistical differences between alternative and traditional ageing systems with different toasting degrees and periods of time, statistical data processing was conducted. The volatile compounds were grouped into chemical families. Visualisations of the differences between the ageing systems were plotted using the R package “ggplot2” with the geom_violin function [22].

To determine which factors had a statistically significant effect on the aroma volatile compounds and aroma series and to evaluate the significance of interactions among factors, a multifactorial analysis of variance was performed by using the type of ageing system, toasting degree, and ripening time as factors.

Multivariate analysis of variance (MANOVA), principal component analysis (PCA), and cluster analysis were performed to determine significant differences between wines aged in three different systems, with two toasting degrees and two ageing times. Data were expressed as mean and standard deviation, and were considered significant at *p* < 0.05. These statistical data analyses were performed using Statgraphics Centurion XVI from StatPoint Technologies Inc. (Warrenton, Virginia).

Orthogonal partial least squares discriminant analysis (OPLS-DA) was performed using the Umetrics SIMCA V14.1 (Umetrics, Sweden) software; only the OAV data with detection threshold over 1 were included. Direct comparison of OAVs between ageing methods (chips, staves, or barrels) at 1.5 and 3 months of ageing was performed using the same software and visualised in a biplot (https://www.sartorius.com/en/products/process-analytical-technology/data-analytics-software/mvda-software/simca (accessed on 2 November 2023)).

## 3. Results and Discussion

### 3.1. Volatile Compounds

**Figure 1:**
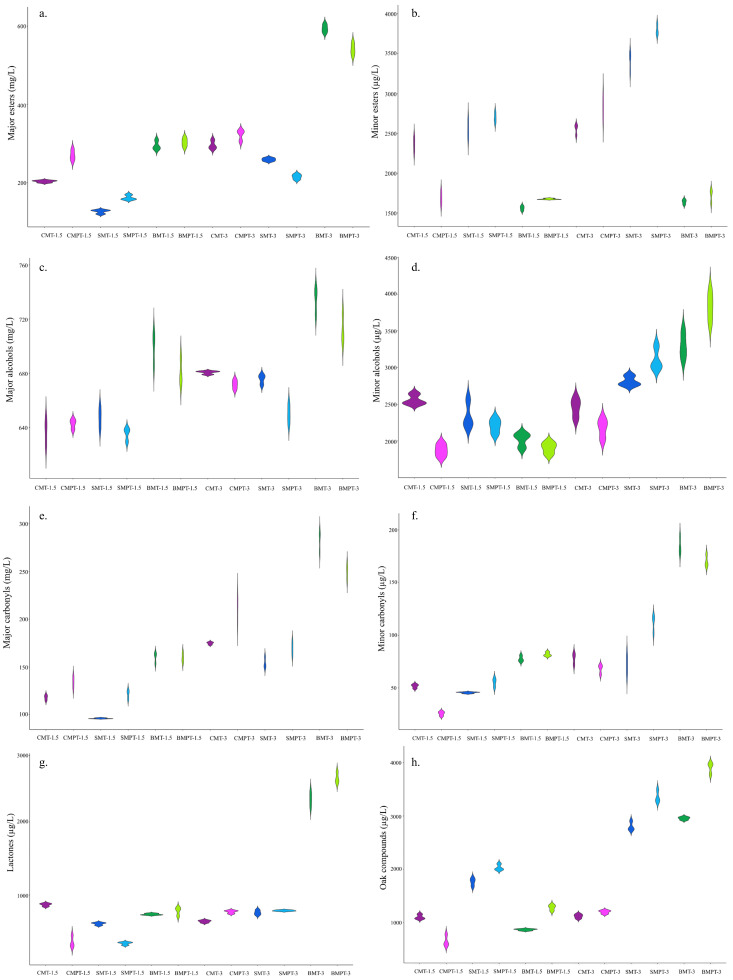
Violin plot with concentrations of chemical families in red wines aged with oak chips, staves, and barrels for 1.5 and 3 months. Dark fuchsia: wine treated with medium toast oak chips; light fuchsia: wine treated with medium plus toast oak chips; dark blue: wine treated with medium toast staves; light blue: wine treated with medium plus toast staves; dark green: wine aged with medium toast barrels; light green: wine aged with medium plus toast barrels. (**a**) Major esters, (**b**) minor esters, (**c**) major alcohols, (**d**) minor alcohols, (**e**) major carbonyls, (**f**) minor carbonyls, (**g**) lactones, and (**h**) oak compounds.

In this study, we identified 42 volatile compounds in wine samples with three types of ageing systems. The volatile compounds detected are major esters (3), minor esters (13), major alcohols (5), minor alcohols (5), major carbonyls (2), minor carbonyls (3), lactones (4), and oak compounds (7), and they are presented in Figure 1a–h. The major volatile compounds are expressed in mg/L, while the minor volatile compounds are grouped by chemical families and expressed in µg/L.

Comparative studies included samples aged with oak chips and staves as representatives of alternative ageing systems and samples aged in barrels as representatives of the traditional system. All three ageing systems used medium toast and medium plus toasted wood for 1.5 and 3 months, respectively.

### 3.2. Evolution of Chemical Families and Aroma Compounds Aged with Alternative and Traditional Systems

#### 3.2.1. Esters

Esters are compounds formed by the condensation of a hydroxyl group of a phenol or alcohol with a carboxyl group from an organic acid [23]. The presence of esters mainly derives from the activity and specificity of β-oxidation enzymes in the fatty acid metabolism pathway, while the hydrolysis of esters may result in the absorption of acids and major alcohols [24]. In wine, more than 150 different esters can be identified, but most of them are present at the trace level [23].

A comparison of samples aged for 1.5 and 3 months in three distinct types of ageing (chips—C; staves—S; and barrels—B) showed that SMPT wines exhibited the highest content of the sum minor esters compared to the CMPT and BMPT. In the case of major esters, the BMT and BMPT held the highest contents after 3 months of ageing (Figure 1a), while in both ageing periods, the BMT and BMPT presented the lowest contents of minor esters in relation to alternative ageing (Figure 1b).

Higher contents of ethyl propionate, ethyl isobutanoate, and ethyl butanoate were found in wines aged in barrels compared to those treated with oak alternatives. On the other hand, the contents of isoamyl acetate, ethyl octanoate, ethyl decanoate, ethyl tetradecanoate, and ethyl hexadecanoate were higher in the wines treated with oak alternatives than in those aged in barrels. Moreover, the contents of ethyl vanillate and ethyl dodecanoate were higher in the wines treated with staves than in the other two types of ageing (Figure 1a,b and Supplementary Table S1). Barrera Garcia et al. [25] showed that the sorption processes are plainly reliant upon the surface of wood that is in touch with the wine, proposing that in conventional barrel ageing, the radial longitudinal surface of the staves in touch with wine displays the lowest macroporosity. Furthermore, Coelho et al. [26] showed that the oak chip particle size influences the time needed to reach the sorption equilibrium of volatile compounds from wine.

During ageing, some ester compounds tend to increase, and their evolution can fluctuate with the conditions of ageing. Ethyl propionate, ethyl butanoate, ethyl octanoate, and ethyl acetate were found above their perception thresholds in all of the wines. In general, esters, with the exception of ethyl acetate, are significant contributors to wine aroma with fruity and floral odours (Table 1). Acetate esters are composed of two main groups: an alcohol group (from ethanol or from a higher alcohol derived from yeast amino acid metabolism) and an acid group (acetate) [27]. These pleasant-odour esters contain isoamyl acetate and ethyl hexanoate, and they are described as having a banana aroma. Phenylethyl acetate is related to rose, fruity, and honey aromas. Ethyl octanoate and ethyl 2-methyl-butanoate are related to pineapple and strawberry aromas, respectively, while ethyl butanoate and ethyl decanoate are related to floral, caramel, nut, and dried fruit aromas [28].

#### 3.2.2. Alcohols

Higher alcohols are produced from the metabolism of amino acids, aldehydes, and sugar during fermentation. During ageing, alcohols react with organic acids to form esters or can be oxidised into aldehydes, resulting in a content decrease [47]. The BMT wines, at both 1.5- and 3-month ageing periods, had higher contents of total major alcohols than the CMT and SMT wines (Figure 1c). Among chips and barrels, the content of major alcohols does not present significant differences. Regarding the minor alcohols, at 1.5 months, no significant differences between the three types of ageing systems were observed. At 3 months, the BMPT wines had the highest content of minor alcohols (Figure 1d).

Some alcohols showed differences between wines aged in barrels and those treated with oak chips and staves, with higher concentrations of propanol, isoamyl alcohol, and 2-phenylethanol being obtained in the wines from barrels. Contrary to this, Rubio-Bretón [48] found a higher content of 2-phenylethanol in wines that developed malolactic fermentation in contact with chips compared to those made in barrels. Also, a difference was found for Z-3-hexenol and E-2-hexenol, with a higher concentration in the alternative system than in the traditional one. Z-3-hexenol is directly related to wine, considering that it is derived from polyunsaturated fatty acids in grapes [49]. The contents of isoamyl alcohol, isobutanol, and 2-phenylethanol were found above their perception thresholds in all of the wines, while the other alcohols, like 1-hexanol and furfuryl alcohol, were found in contents below their threshold levels. 2-Phenylethanol is an alcohol with a positive aroma due to rose notes [50]. Also, many higher alcohols possess pleasant aromas, such as active amyl alcohol or isoamyl alcohol, with a marzipan aroma [23]. It was found that higher contents of isoamyl alcohol and isobutanol can be related to a transformation of the amino acid catabolism induced by plant abiotic stress [51]. 1-Hexanol has imparted a green, grassy, and fresh odour [52] (Table 1).

#### 3.2.3. Carbonyls

Carbonyls are formed by the oxidation of fatty acids and higher alcohols, Strecker degradation, aldol condensation, or Maillard reactions [53]. Carbonyl compounds are predominantly products of yeast and bacterial metabolism. Therefore, their highest contents in wine occur immediately after fermentation. Some may be responsible for off-flavours, while others, with pleasant aromas, were identified as important contributors to wine aroma.

For both the 1.5- and 3-month ageing periods, the traditional barrel system presented a higher content of major carbonyls when compared with the alternative system of ageing (Figure 1e). In the case of minor carbonyls, after 3 months of ageing, the BMT had the highest content (Figure 1f). Thus, for major and minor carbonyls, the barrel samples present the same behaviour, having the highest content. In the group of carbonyl compounds, differences in the content were observed in acetaldehyde, acetoin, octanal, and benzaldehyde, which reached higher values in the wines aged in barrels than in those treated with oak chips and staves. Acetaldehyde plays an important role in wine aroma; this depends on its contents. Thus, at high values, like >200 mg/L, it provides platitude to the wines, but at low levels, it gives off fruity aromas.

#### 3.2.4. Lactones

Lactones are aroma compounds commonly containing a five- (γ-) or six-membered (δ-lactone) cyclic ester, while furanones contain a five-membered heterocyclic ring [54]. Lactones are associated with a variety of aroma characteristics, such as coconut, fruity, sweet, caramelly, peachy, apricot-like, dried fruit, spicy-green, fatty, and oily characteristics, and they have a positive impact on the aroma profile [55]. At 1.5 months, CMPT and SMPT had the lowest values compared with CMT and SMT. At 3 months, CMT, CMPT, and SMT had similar values, while those of BMT and BMPT increased by 3-fold in rapport with the anterior variants (Figure 1). A high amount of γ-butyrolactone was determined in all of the samples, especially in BMPT after 3 months of ageing (Figure 1g and Supplementary Table S1). Hevia et al. [56] found similar results with high concentrations of this compound in aged oloroso wines.

In the group of lactones, differences in the contents of γ-crotonolactone and γ-butyrolactone were noticed after 3 months in the wines aged in barrels compared with the wines treated using alternative systems. These aforementioned lactones were found below their perception thresholds in all of the wines. γ-Crotonolactone and γ-butyrolactone present positive aromas due to toasty, sweet, and caramel notes (Table 1). γ-Nonalactone was a potent odourant contributing stone fruit aromas in dry white and botrytised wines [57]. Also, γ-nonalactone has been identified in connection with the dried fruit aromas in red wines subjected to premature ageing or wines made from overripe or dehydrated grapes [55].

#### 3.2.5. Oak Compounds

Oak compounds are among the most important aromas contributing to the sensory characteristics of wines aged with oak wood. They are naturally present in oak wood, and their concentrations in wines increase during the ageing process. Furthermore, they have been reported as potential ageing markers in red wines. At 1.5 months, CMPT exhibited the lowest values, and SMPT had the highest values. At 3 months, CMT presented the lowest values, while SMPT and BMPT presented the highest values. The values of the stave samples with medium plus toast were close to those of the barrel sample with the same type of toasting (Figure 1h and Supplementary Table S1).

In the case of oak compounds, cis-whisky lactones present higher contents in wines treated with staves than those treated with oak chips and barrels (Supplementary Table S1). Furfural and guaiacol reached higher concentrations in the wines aged in barrels than in the wines with added alternatives of oak after 3 months of ageing. These results were in agreement with other studies [58,59], in which the highest amounts were found in the traditional aged spirits when compared with those proceeding from the alternative ageing system, using staves without micro-oxygenation. The content of the cis isomer increases over time. The content of the cis isomer was above its perception in the stave and barrel samples, while furfural and 5-methylfurfural were below their perception thresholds in all samples. Caldeira et al. [60,61] found that the values of the cis isomer of β-methyl-γ-octalactone were higher in the wine spirits aged through the traditional system. Also, Caldeira et al. [62] found that this compound was higher in wine spirits aged using the alternative system.

In general, the contents of volatile compounds extracted from wood ageing increased significantly during the process. Medium-toasted casks produce wines with the greatest volatile compositions [56,63]. The same behaviour was presented by guaiacol, a compound derived from the degradation of lignin. Although its content increased slowly in all of the aged wines regardless of the system of wood type used, guaiacol presented the highest content when aged in BMPT. Furfural and 5-HF increased considerably with ageing, while 5-HMF increased to a lesser degree.

The alternative ageing system changes the number of volatile compounds. Firstly, these differences result from the effects of the toasting process on the wood. The oven heating process induces a higher level of various furanic compounds, phenol derivates, and oak compounds on the staves than in the barrels. Secondly, the concentration of the most volatile compounds increased when the wines were treated with staves. Thus, the principle of Fick’s Law was in accordance with our study. An increase in surface area for molecular transport contributes to an enhanced mass transfer of these compounds. Thirdly, the compounds showed statistically significant differences between the two ageing periods, and after 3 months of ageing, the concentrations of these compounds were higher than they were at 1.5 months.

### 3.3. Odour Activity Values (OAVs)

Table 2 presents changes in the odour activity values (OAVs) of the volatile compounds in wines aged at 1.5 and 3 months with chips, staves, and barrels. The OAVs were calculated by dividing the concentration of each volatile compound by its respective detection threshold [64]. The OAV is another indicator that can determine the intensity of a certain substance’s aroma contribution. Substances with OAVs ≥ 1 are generally considered characteristic aroma substances that contribute to the overall aroma of the sample [65].

A total of 22 aroma-active compounds, with OAV > 1, were detected in red wines aged in different winemaking systems. The aroma-active compounds for red wines depend on their concentrations and on the odour threshold values (Table 2). The OAV results revealed the highest values for isoamyl acetate (OAV 11.61–66.26), ethyl propionate (OAV 18.46–56.41), ethyl butanoate (OAV 10.28–27.37), ethyl octanoate (OAV 1.80–17.37), isoamyl alcohols (OAV 8.74–9.92), 2-phenylethanol (OAV 6.27–9.32), and cis-whiskey lactone (OAV 0.11–7.43). Lower OAVs were observed for octanal (OAV 0.44–7.30), 2-methoxy-4-vinylphenol (OAV 0.38–2.13), guaiacol (OAV 0.10–1.08), and γ-nonalactone (OAV 0.69–1.52).

The extraction of furfural and 5-methylfurfural increased over time, but they showed exceedingly low OAVs (Table 2). This increase suggests that the extraction of lactones from their glycoconjugates could have taken place using the two alternative methods (oak chips and staves) and the traditional method (barrel) during ageing. Lactones are responsible for the “burned almonds”, “caramel”, and “nutty-like” aromas conferred on oak-aged wines and are considered, based on their relative odour intensities, the most impactful oak-derived aromas [66]. Cis-whisky lactone showed increased OAVs in all of the wines at 3 months, especially in the wines aged with staves (OAV-7.431), implying its relevance in the sensory composition of the analysed wine samples. Trans-whisky lactone is often found in wines at relatively lower values than its cis counterpart and presents low OAVs; therefore, it is unlikely to be of practical relevance. In French oak barrels of different origins, the ratios of the cis/trans lactone isomers have been revealed to be 1.3, 1.4, 1.6, and 1.7 [67,68]. In our study, the ratio of cis/trans lactone isomers after 3 months of ageing for BMT was 1.99, and for BMPT, it was 2.3. The OAVs of cis-whiskey lactone are responsible for the typical “woody, coconut notes, and vanilla” aromas.

The volatile compounds evolved in the two time periods among the three ageing systems are investigated in this article. The wood-related compounds showed differences among ageing using barrels and chips or staves. After 3 months of ageing, the wines in barrels maintained the higher concentrations than the ones aged for 1.5 months. In this study, we observed increases in 2-phenylethanol, acetoin, octanal, ethyl propionate, ethyl isobutanoate, ethyl butanoate, ethyl lactat, guaiacol, and 2-methoxy-4-vinylphenol in the wines aged in barrels compared with wines aged with alternative chips or stave samples. Interestingly, we noticed decreases in E-3-hexenol, heptanal, isoamylacetate, ethyl octanoate, ethyl decanoate, and cis-whisky lactone in the wines aged in barrels at 3 months. Moreover, we were able to detect ethyl cinnamate only in the wine samples aged in barrels for 3 months (Table 2). Alcohols, esters, and oak compounds were the main contributors to the samples’ aroma profiles, and their OAVs were higher in the stave samples after 3 months than in the other two systems of ageing. Isoamyl alcohols and 2-phenylethanol were the most pronounced alcohols in the wine samples. Both compounds depended on the ageing type and time factors; however, isoamyl alcohols were not influenced by the toasting degree. In the samples aged in barrels, after 3 months, ethyl propionate showed high levels, whereas in the samples treated with staves, it seems that it was derived from isoamyl acetate and ethyl octanoate. Table 2 presents the MANOVA for OAV calculated for each volatile compound of the 1.5- and 3-month ageing processes of wines using three different methods. Significant effects of the type of ageing system were observed in almost all analysed volatile compounds, with the exception of ethyl isobutanoate and ethyl dodecanoate.

No major differences were found in the total OAV of traditional and alternative systems for isobutanol, acetaldehyde, phenylethyl acetate, ethyl acetate, and nonalactone. Our results indicate that wine aged for 3 months in barrels, chips, or staves maintain high OAVs for major aroma compounds. However, wines aged with staves showed the highest chemical compound concentrations and had more complex aromatic profiles based on OAVs than the wines aged in barrels or with chips.

### 3.4. Aromatic Series

Eleven aromatic series of aged red wines based on OAVs were observed, as shown in Table 3. The aged red wine samples were characterised mainly by fruity, chemistry, waxy, floral, spicy, and woody series. Interestingly, the aromatic series showed differences among the ageing systems. The SMPT-3 (>225) and CMPT-3 (>170) wines showed the highest levels of aromatic series, suggesting that alternative systems provided a more pronounced wood aroma than the traditional system (BMPT-3 > 150). The fruity series showed a higher intensity in the samples treated with medium plus toast staves (>160) and oak chips (>120) after 3 months of ageing; the chemistry series showed higher levels in the barrel samples at 3 months of ageing (>20); and the woody series showed higher levels in the medium toast staves (>7). In addition, the wines aged in all three systems showed low odour values for honey and citrus (<1), which suggests that these types of flavours do not regularly influence the toasting levels and cannot be perceived by consumers.

Active odourants are the direct components of the aromatic series. The global aroma attributes were calculated by summing each volatile in each given aroma series. The fruity aroma was the dominant series, followed by the chemistry, waxy, and floral series. Spice, woody, and caramel aromas were also considered the featured aromas for the aged wines. An increasing trend in the fruity aroma was observed in all systems of ageing after 3 months. The highest values were in the staves compared to those of the chips and barrels. Barrel ageing possessed the lowest green aroma character of chips and staves throughout the two periods investigated. A significant difference was observed in the case of the waxy series, where the chips and staves presented high values compared with the barrels. The woody and spice series increased with the ageing time, and the highest values were in the wines aged with medium toast staves. In contrast, the wine aged with medium plus oak chips had the lowest values for the same series. Overall, all investigated series increased with the ageing time in both the alternative and traditional systems. The majority of aromatic series depend on the type, toasting, and time factors. An exception is made for the green and fruity series, which do not depend on the toasting factor. Also, the honey series does not depend on any of the investigated factors.

Isoamyl acetate, ethyl propionate, ethyl butanoate, and ethyl octanoate were the primary contributors to the fruity aroma; the chemistry aroma was produced mostly by isoamyl alcohols, ethyl acetate, isobutanol, guaiacol, and other compounds. The spice and woody series were contributed mainly by trans- and cis-whiskey lactone, with mention that in the first series, there was one more contributor, the ethyl cinnamate. The waxy series was produced by the esters (ethyl octanoate, ethyl decanoate, ethyl dodecanoate, ethyl tetradecanoate, and ethyl hexadecanoate).

**Figure 2:**
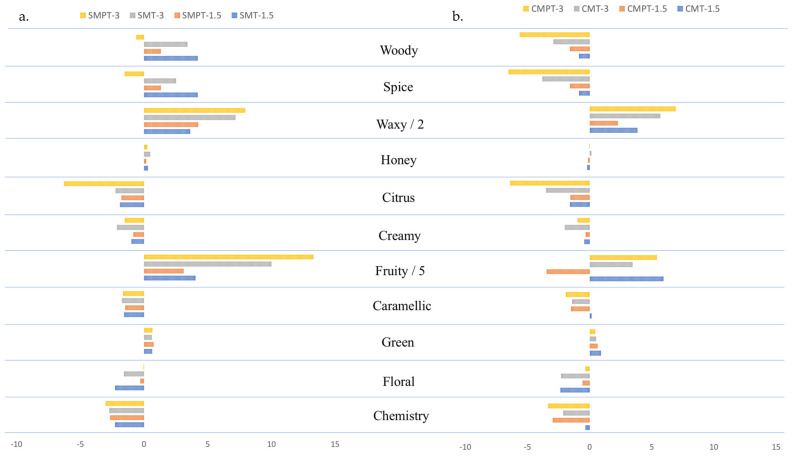
Aromatic series differences among wines aged with staves (**a**) and oak chips (**b**) in relation to the wines aged in barrels.

The differences in aromatic series values between alternative ageing systems (staves and oak chips samples) in relation to the traditional system (barrel samples) at 1.5 and 3 months are shown in Figure 2. Positive values indicate an increase in the aromatic series of staves (a) and chips (b) with respect to the wines aged in barrels, while negative values indicate a decrease in the studied aromatic series. The values for each sample are presented in Table 2. It is obvious that the system of ageing produced an important change in the aromatic series, represented by positive and negative values in the graphs. Greater differences, in respect to the barrel samples, were found for the fruity series, especially in the wines treated with staves after 3 months (SMPT-3) (Figure 2a). The fruity series had 66 positive differences in the samples treated with medium plus toasted staves when compared with the barrel samples. In general, the woody, waxy, spicy, honey, and green series presented positive values and were higher in the stave samples than in the barrel samples. For the woody and spicy series, the highest values were identified in SMT-1.5. Also, for the same aromatic series previously mentioned, we can observe that only SMPT-3 has negative values. The rest of the series, including citrusy, creamy, caramelly, floral, and chemistry, in the samples aged with staves have negative values and are the lowest in rapport with the samples aged in barrels.

In Figure 2b of the oak chip samples, only the fruity, waxy, and green series have positive values and are bigger than those of the samples aged in barrels. Also, the fruity series maintained the highest values of all series, with 29 positive differences in the samples treated with medium toast oak chips after 1.5 months. An exception was registered for medium plus toast oak chips aged for 1.5 months, which had negative values. The woody, spicy, citrusy, creamy, caramel-like, floral, and chemistry series had negative values in the samples treated with oak chips in relation to the barrel samples. A positive trend can be observed in Figure 2 for the chemistry, floral, caramelly, creamy, and citrusy series that presented positive values in the traditional system and negative values in the alternative samples. These findings suggest that high values for the aforementioned aromatic series were found in the barrel samples and lower values were found in the stave and oak chip samples. The fruity, spicy, and woody series had negative values in the barrel and oak chip samples, while in the stave samples, these series were positive. The wines aged with staves had a stronger fruity, spicy, and woody aroma, while the samples aged in barrels presented a more chemistry-driven, floral, caramelly, and creamy aroma. This type of wood (staves) is the most suitable of the three types in this study and also contains the greatest amount of cellulose, which supports the formation of these compounds during the toasting of the wood and their extraction during the ageing process.

Studies on the impacts of the time period and sensory properties of wine suggest that the use of alternative systems, like chips and staves, in comparison with barrels can lead to an increase in some chemical compounds, such as furfural [69]. However, authors observed that wine exposed to oak products for a longer time, e.g., more than 6 months, could result in a decrease in furan aldehydes, as they are transformed to the corresponding alcohols. The loss of 5-methylfurfural concentration was even more pronounced, showing a decrease after 3 months of ageing. In the same study, wines matured in oak barrels showed increased concentrations of oak compounds, relative to the duration of ageing process, in comparison with the chip samples. In our study, we noticed a clear increase in concentrations for all chemical classes investigated between 1.5 and 3 months of ageing. However, further investigations are necessary to evaluate the concentrations for chemical classes in regard to the duration of the alternative ageing process, such as 6 months and more.

### 3.5. Principal Component Analysis (PCA) and Orthogonal Partial Least Squares Discriminant Analysis (OPLS-DA)

In order to evaluate if the variables could help to discriminate between the ageing system or toasting degree or time of ageing, a principal component analysis (PCA) was applied to the 22 OAVs above 1. A principal component analysis (PCA) is a multivariate data analysis technique used for dimensionality reduction and for showing relationships or correlations between the variables and samples [70].

**Figure 3:**
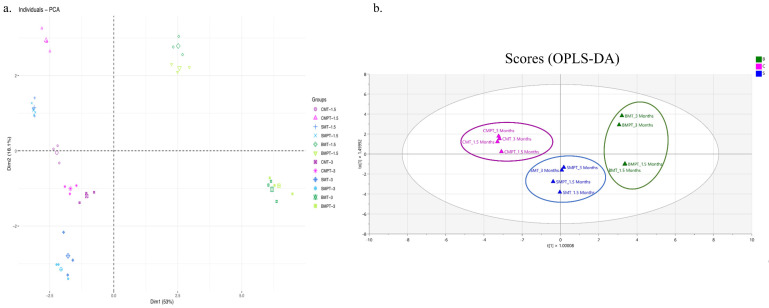
(**a**) Principal component analysis using odour activity values above the unity as classifying variables. CMT and CMPT: wines treated with medium toast or medium plus toast chips. SMT and SMPT: wines treated with medium toast or medium plus toast staves. BMT and BMPT: wines aged in medium toast or medium plus toast barrels. The values 1.5 and 3 point to the months of ageing. (**b**) Orthogonal partial least square data analysis (OPLS-DA) for the discrimination of wine based on ageing method. The second component, which accounts for only 18.1% of the total variance, contributes to a slight splitting between the samples according to the ageing time. Wines aged at 1.5 months are located on the positive side, while samples aged for 3 months are located on the negative side. The variables with the highest contribution on the negative side are ethyl octanoate and nonalactone, while on the positive side, the variables with the higher loading vector are ethyl isobutanoate and cis-whiskey lactone, confirming that this is a good marker for ageing discrimination among samples. The results of the PCA suggest that it is possible to use OAVs and discriminate red wine according to the ageing system.

The first two main components comprise 71.1% of the total accumulated variance, as presented in Figure 3a. The first component represents 53% of the total variance and separates the types of ageing systems. The samples aged in the traditional system were located on the positive side of the axis. This is associated with the odour values of the volatile compounds extracted from the wood, like guaiacol, 2-phenylethanol, octanal, isoamyl alcohol, ethyl cinnamate, and ethyl lactate. The samples aged in medium toast and medium plus toast barrels for 3 months had richer and more diverse odour activities of volatile compositions than those aged for 1.5 months. This greater richness may be due to the longer residence time in the barrel, during which the slow extraction, oxidation, or condensation reactions occur. The samples of wine from the alternative system were located on the opposite side of component 1, and they are connected with the odour values existing in red wines, such as heptanal, ethyl decanoate, and isoamyl acetate.

OAVs were used for a discrimination analysis among ageing methods using OPLS-DA. The results showed that wine samples could be easily clustered based on ageing technique, regardless of the toasting method and ageing time used (Figure 3b). In conclusion, both investigation methods could discriminate among ageing techniques; however, OPLS-DA could be used for a clearer classification of wine ageing in traditional or alternative systems in comparison to PCA-based classification.

**Figure 4:**
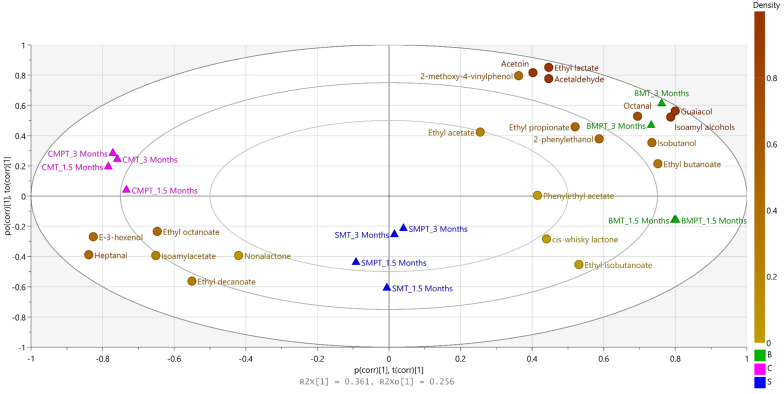
OPLS-DA analysis of nine wine varieties. Note, ageing method in triangles: C: chips, S: staves, B: barrels, MT: medium toast, MPT: medium plus toast. OAVs in coloured dots are based on overall mean density on a scale from 0 to 1.

OPLS-DA is a statistical method that permits the discrimination of differences among analysed samples in terms of the OAVs and ageing methods, e.g., chips, staves, or barrels. Moreover, this method enables the identification of markers of samples investigated [71,72]. We used the content of OAVs to investigate the overall influence of wine ageing technique and flavour, as previously described in the literature by Cao et al., 2022 [73] and Jin et al., 2021 [74]. The human perception of the OAV smell corresponds to values > 1, resulting in 22 compounds that were used for the OPLS-DA model. The OAVs were used as Y variables and the ageing methods were used as X variables for the OPLS-DA investigation in order to observe different contributions of volatile compounds to ageing methods (Figure 4).

As expected, the wine aroma quality is influenced by a combined effect of the ageing method and compound intensity. The results indicate that among the major contributing compounds for wines aged with chips at 1.5 or 3 months are E-3-hexenol, heptanal, isoamylacetate, ethyl octanoate, ethyl decanoate, and nonalactone. On the other hand, wines aged in barrels have pregnant aroma profiles generated by all other OAVs included in this analysis. Interesting, wines with staves are located in the middle, suggesting that all compounds contribute in various amounts to the final aroma profiles of these wines.

### 3.6. Cluster with Aroma Compounds

**Figure 5:**
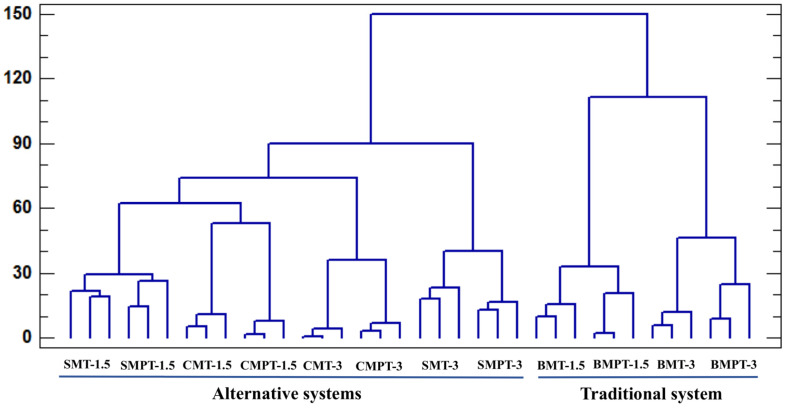
Cluster analysis using as variables all volatile compounds identified in aged wine samples.

A cluster analysis was carried out in order to assess the similarities between the different ageing systems, using the concentrations of all volatile compounds as classifying variables (Supplementary Table S1). The similarity was higher when the distance between two clusters was smaller. In Figure 5, we can observe two main clusters that differentiate the wine samples according to the type of ageing system.

In the first cluster, the samples treated with oak chips and staves were grouped into alternative systems. These formed distinctive clusters according to ageing time and the type of system used. The occurrence of these samples in the same cluster fits their similar winemaking process and illustrates the similarity between the two. Overall, volatile fingerprinting allowed for the identification of trends from a dataset that was coherent with the differences between the samples studied. The second cluster was formed by two subclusters, thus differentiating the samples aged in barrels for 1.5 months with those aged for 3 months (Figure 5). These results indicate that the type of ageing system and ageing time represent key factors in the aroma compounds.

## 4. Conclusions

Odour activity values are important tools for the classification of ageing systems in red wine production. A principal component analysis using odour activity values showed a clear relationship between the wine samples. The analytical results indicate that alcohols, esters, and oak compounds were the main contributors to the wine aroma profile. The OAVs were higher for these major aroma compounds in the stave samples after 3 months than in the other two systems of ageing. Wines aged with staves have a stronger fruity, spicy, and woody aroma, while samples aged in barrels present a more chemistry, floral, caramelly, and creamy aroma. SMPT-3 and CMPT-3 generated the highest levels of aromatic series, suggesting that alternative systems provide stronger aroma profiles than traditional systems after 3 months of ageing (BMPT).

The PCA results indicate that it is possible to discriminate wine according to the ageing system. PCs are associated with the odour values of the volatile compounds extracted from the wood, like guaiacol, 2-phenylethanol, octanal, isoamyl alcohol, and ethyl cinnamate. Wine samples aged with the alternative system are on the opposite side of PC1 and comprise heptanal, ethyl decanoate, and isoamyl acetate. Similar results were observed for the discrimination analysis using OPLS-DA.

Generally, our results indicate that the use of staves could be an easy-to-use alternative to barrels for red wine ageing. Staves may represent a fast treatment to improve wine aroma and could increase the sustainability of the entire winemaking process. Moreover, the volatile fingerprint proved to be suitable for the analysis and detection of differences in wine composition, clearly reflecting changes in production processes, and it is thus a promising tool for monitoring winemaking and ageing processes.

## Figures and Tables

**Table 1:** Odour descriptor, odour threshold, and aroma series assigned to the volatile compounds identified in the wines analysed.

Compounds	Formula	CAS ^a^	Odour Descriptor	Odour Threshold ^b^ (μg/L)	Aroma Series ^c^
Methanol	CH_4_O	67-56-1	Chemical, medicinal, fruity, pungent	668,000 [29]	1
Propanol	C_3_H_8_O	71-23-8	Ripe fruit, fusel alcohol	830,000 [30]	1
Isobutanol	C_4_H_10_O	78-83-1	Nail polish, bitter	40,000 [30]	1
Isoamyl alcohols	C_5_H_12_O	123-51-3	Burnt, alcohol, nail polish, whisky, ripe fruit	30,000 [30]	1
2-phenylethanol	C_8_H_10_O	60-12-8	Rose, honey, lilac	10,000 [30]	2
Hexanol	C_6_H_14_O	111-27-3	Green, grass, oily	8000 [30]	3
E-3-hexenol	C_6_H_12_O	928-97-2	Green, grass	400 [31]	3
E-2-hexenol	C_6_H_12_O	928-95-0	Green tomato	15,000 [32]	3
Furfuryl alcohol	C_5_H_6_O_2_	98-00-0	Paint, burnt, coffee	8000 [30]	1, 4
Benzyl alcohol	C_7_H_8_O	100-51-6	Floral, phenolic, sweet	200,000 [33]	2, 5
Acetaldehyde	C_2_H_4_O	75-07-0	Pungent, ripe apple	110,000 [30]	1, 3
Acetoin	C_4_H_8_O_2_	513-86-0	Yogurt, butterscotch	150,000 [30]	6
Heptanal	C_7_H_14_O	111-71-7	Herbal, coriander	3 [34]	3
Octanal	C_8_H_16_O	124-13-0	Citrus, green, fresh	2.5 [35]	7
Benzaldehyde	C_7_H_6_O	100-52-7	Bitter almond, smoked, walnut	350 [34]	5
Ethyl propionate	C_5_H_10_O_2_	105-37-3	Fruity, grape, pineapple	10 [36]	5
Ethyl isobutanoate	C_6_H_12_O_2_	97-62-1	Ripe melon, apple, strawberry	15 [30]	5
Ethyl butanoate	C_6_H_12_O_2_	105-54-4	Fruity, floral, sweet, apple	20 [37]	5
Isoamyl acetate	C_7_H_14_O_2_	123-92-2	Banana	30 [38]	5
Ethyl octanoate	C_10_H_20_O_2_	106-32-1	Banana, pineapple, floral, pear, soapy	5 [38]	5, 9
Phenylethyl acetate	C_10_H_12_O_2_	103-45-7	Fruity, honey, floral	250 [37]	8
Ethyl decanoate	C_12_H_24_O_2_	110-38-3	Sweet, fruity, caramel, nuts, and dried fruit	200 [38]	5, 9
Ethyl tetradecanoate	C_16_H_32_O_2_	124-06-1	Waxy, buttery, fatty odour	4000 [30]	9
Ethyl vanillate	C_10_H_12_O_4_	617-05-0	Smoky, burnt	990 [33]	1, 6
Ethyl dodecanoate	C_14_H_28_O_2_	106-33-2	Creamy, floral	500 [30]	9
Ethyl hexadecanoate	C_18_H_36_O_2_	628-97-7	Fatty, rancid, fruity, sweet, caramel	2000 [30]	9
Phenethyl phenyl acetate	C_16_H_16_O_2_	102-20-5	Floral, honey	250 *	2, 8
Ethyl cinnamate	C_11_H_12_O_2_	4610-69-9	Fruity, soapy, cinnamon-like, spice	1.1 [39]	10
Ethyl acetate	C_4_H_8_O_2_	141-78-6	Fruity, glue, pineapple, varnish, balsamic	7500 [30]	1
Ethyl lactate	C_5_H_10_O_3_	97-64-3	Lactic, yogurt, buttery	150,000 [40]	6
Diethyl succinate	C_8_H_14_O_4_	123-25-1	Overripe melon	200,000 [33]	5
Crotonolactone	C_4_H_4_O_2_	497-23-4	Toasty, buttery	35,000 [41]	6
Butyrolactone	C_4_H_6_O_2_	96-48-0	Sweet, caramel	35,000 [42]	6
Nonalactone	C_9_H_16_O_2_	6008-27-1	Coconut, creamy	30 [39]	5, 6
Decalactone	C_10_H_18_O_2_	706-14-9	Peach, milky	47 [39]	5
*trans*-whisky lactone	C_9_H_16_O_2_	39638-67-0	Woody, coconut notes, vanilla	370 [43]	10, 11
*cis*-whisky lactone	C_9_H_16_O_2_	55013-32-6	Woody, coconut notes, vanilla	54 [43]	10, 11
Guaiacol	C_7_H_8_O_2_	90-05-1	Medicine, smoke, woody	75 [44]	1, 4
2-methoxy-4-vinylphenol	C_9_H_10_O_2_	7786-61-0	Spices, clove, woody	40 [45]	1, 4
Furfural	C_5_H_4_O_2_	98-01-1	Burned almonds, caramel, nutty	770 [46]	4
5-methylfurfural	C_6_H_6_O_2_	620-02-0	Caramel, roasted almond, loral, sweet, and bready	1100 [46]	4
5-HMF	C_6_H_6_O_3_	67-47-0	Caramel, butter, waxy, tobacco	8000 [13]	4

CAS ^a^: Chemical Abstract Service. Odour threshold ^b^ (μg/L): expressed as phenylethyl acetate. Aroma series ^c^: 1. Chemistry; 2. Floral; 3. Green; 4. Caramelly; 5. Fruity; 6. Creamy; 7. Citrus; 8. Honey; 9. Waxy; 10. Spicy; 11. Woody. * expressed as phenylethyl acetate.

**Table 2:** Odour activity values (OAVs) detected in wines aged with oak chips, staves, and barrels for 1.5 and 3 months. Multivariate analysis of variance (MANOVA) was conducted to study the influence of the type of ageing system (AS), the toast degree (TG), the ageing time (AT), and the interaction among factors (In) in the volatile aroma compound concentration. C: chips; S: staves; B: barrels; MT: medium toast; MPT: medium plus toast.

Compounds	CMT	CMPT	SMT	SMPT	BMT	BMPT	CMT	CMPT	SMT	SMPT	BMT	BMPT	MANOVA			
	1.5 Months						3 Months						AS	TG	AT	In
Methanol	0.329 ± 0.01	0.343 ± 0.00	0.339 ± 0.00	0.338 ± 0.01	0.346 ± 0.01	0.343 ± 0.02	0.369 ± 0.01	0.372 ± 0.00	0.357 ± 0.00	0.341 ± 0.01	0.379 ± 0.01	0.368 ± 0.02	**	ns	***	ns
Propanol	0.043 ± 0.00	0.044 ± 0.00	0.044 ± 0.00	0.044 ± 0.00	0.052 ± 0.00	0.054 ± 0.00	0.046 ± 0.00	0.046 ± 0.00	0.046 ± 0.00	0.045 ± 0.00	0.054 ± 0.00	0.057 ± 0.00	***	*	***	ns
Isobutanol	1.170 ± 0.03	1.150 ± 0.02	1.206 ± 0.01	1.197 ± 0.02	1.220 ± 0.01	1.243 ± 0.02	1.226 ± 0.02	1.206 ± 0.03	1.216 ± 0.02	1.200 ± 0.03	1.249 ± 0.01	1.273 ± 0.02	***	ns	***	ns
Isoamyl alcohols	8.850 ± 0.13	8.945 ± 0.07	8.996 ± 0.08	8.738 ± 0.23	9.464 ± 0.09	9.600 ± 0.18	9.212 ± 0.18	8.985 ± 0.07	9.229 ± 0.12	9.053 ± 0.15	9.793 ± 0.10	9.916 ± 0.20	***	ns	***	ns
2-phenylethanol	6.901 ± 0.06	6.276 ± 0.22	6.567 ± 0.49	6.265 ± 0.08	9.264 ± 0.89	6.848 ± 0.36	7.004 ± 0.08	6.711 ± 0.13	7.297 ± 0.42	6.526 ± 0.18	9.316 ± 0.34	7.072 ± 0.37	***	***	*	ns
Hexanol	0.226 ± 0.01	0.166 ± 0.01	0.215 ± 0.02	0.201 ± 0.01	0.201 ± 0.01	0.180 ± 0.01	0.222 ± 0.01	0.169 ± 0.01	0.245 ± 0.01	0.279 ± 0.01	0.273 ± 0.02	0.333 ± 0.02	***	**	***	***
E-3-hexenol	0.586 ± 0.01	0.346 ± 0.01	0.365 ± 0.02	0.386 ± 0.02	0.012 ± 0.00	0.004 ± 0.00	0.426 ± 0.03	0.431 ± 0.02	0.465 ± 0.01	0.560 ± 0.03	0.036 ± 0.00	0.045 ± 0.00	***	ns	***	***
E-2-hexenol	0.031 ± 0.00	0.022 ± 0.00	0.027 ± 0.00	0.025 ± 0.00	0.023 ± 0.00	0.025 ± 0.00	0.028 ± 0.00	0.035 ± 0.00	0.039 ± 0.00	0.039 ± 0.00	0.027 ± 0.00	0.033 ± 0.00	***	ns	***	**
Furfuryl alcohol	0.006 ± 0.00	0.009 ± 0.00	0.008 ± 0.00	0.007 ± 0.00	0.007 ± 0.00	0.011 ± 0.00	0.007 ± 0.00	0.011 ± 0.00	0.007 ± 0.00	0.005 ± 0.00	0.087 ± 0.00	0.081 ± 0.00	***	***	***	***
Benzyl alcohol	0.000 ± 0.00	0.000 ± 0.00	0.000 ± 0.00	0.000 ± 0.00	0.000 ± 0.00	0.000 ± 0.00	0.000 ± 0.00	0.000 ± 0.00	0.000 ± 0.00	0.000 ± 0.00	0.000 ± 0.00	0.000 ± 0.00	***	*	***	ns
Acetaldehyde	0.470 ± 0.02	0.523 ± 0.01	0.384 ± 0.01	0.479 ± 0.02	0.575 ± 0.02	0.546 ± 0.02	0.565 ± 0.03	0.613 ± 0.08	0.537 ± 0.04	0.621 ± 0.02	0.810 ± 0.03	0.699 ± 0.03	***	ns	ns	ns
Acetoin	0.441 ± 0.01	0.511 ± 0.04	0.358 ± 0.01	0.459 ± 0.02	0.646 ± 0.04	0.663 ± 0.02	0.751 ± 0.03	0.957 ± 0.05	0.633 ± 0.02	0.674 ± 0.04	1.291 ± 0.07	1.152 ± 0.04	***	**	***	**
Heptanal	0.567 ± 0.03	0.544 ± 0.02	0.600 ± 0.07	0.600 ± 0.07	0.159 ± 0.03	0.208 ± 0.06	0.600 ± 0.03	0.556 ± 0.02	0.633 ± 0.03	0.500 ± 0.03	0.178 ± 0.03	0.248 ± 0.02	***	**	***	***
Octanal	0.725 ± 0.02	1.120 ± 0.08	0.440 ± 0.04	0.896 ± 0.08	2.333 ± 0.14	2.679 ± 0.23	0.440 ± 0.04	0.889 ± 0.08	1.720 ± 0.32	1.000 ± 0.08	3.956 ± 0.36	7.299 ± 0.54	***	**	***	ns
Benzaldehyde	0.138 ± 0.01	0.061 ± 0.01	0.122 ± 0.00	0.146 ± 0.01	0.204 ± 0.01	0.213 ± 0.1	0.215 ± 0.02	0.183 ± 0.01	0.188 ± 0.02	0.308 ± 0.02	0.498 ± 0.02	0.433 ± 0.02	***	ns	***	**
Ethyl propionate	35.136 ± 0.3	18.461 ± 1.1	28.442 ± 2.2	28.076 ± 1.9	32.519 ± 2.3	36.829 ± 0.9	34.938 ± 1.3	34.633 ± 0.9	48.195 ± 1.5	51.042 ± 1.2	56.408 ± 2.0	54.500 ± 3.5	***	*	***	***
Ethyl isobutanoate	0.313 ± 0.02	0.476 ± 0.03	0.800 ± 0.00	0.933 ± 0.00	1.313 ± 0.14	1.287 ± 0.10	0.023 ± 0.00	0.102 ± 0.01	0.033 ± 0.00	0.078 ± 0.01	0.342 ± 0.03	0.327 ± 0.01	ns	ns	ns	ns
Ethyl butanoate	17.237 ± 1.1	10.278 ± 0.3	16.238 ± 0.3	16.777 ± 0.7	23.781 ± 0.8	26.736 ± 1.1	19.886 ± 0.4	18.132 ± 0.9	22.538 ± 1.1	26.604 ± 0.9	24.307 ± 1.3	27.372 ± 1.3	***	***	***	***
Isoamylacetate	42.950 ± 2.53	30.536 ± 2.2	41.430 ± 3.5	42.939 ± 2.3	15.944 ± 0.3	16.278 ± 0.4	44.021 ± 1.8	54.086 ± 4.1	57.928 ± 2.5	66.259 ± 1.5	11.667 ± 0.5	11.611 ± 0.7	***	ns	***	*
Ethyl octanoate	9.202 ± 0.39	6.033 ± 0.33	8.288 ± 0.36	9.150 ± 0.46	1.853 ± 0.11	1.800 ± 0.09	12.875 ± 0.2	15.482 ± 0.2	15.582 ± 0.8	17.323 ± 0.5	2.147 ± 0.06	2.167 ± 0.06	***	***	***	***
Phenylethyl acetate	0.316 ± 0.02	0.403 ± 0.01	0.411 ± 0.02	0.421 ± 0.02	0.533 ± 0.03	0.541 ± 0.02	0.508 ± 0.01	0.479 ± 0.02	0.408 ± 0.01	0.468 ± 0.02	0.370 ± 0.00	0.535 ± 0.03	***	***	***	***
Ethyl decanoate	0.417 ± 0.03	0.313 ± 0.02	0.629 ± 0.03	0.782 ± 0.03	0.128 ± 0.01	0.120 ± 0.01	0.615 ± 0.02	0.512 ± 0.02	0.822 ± 0.04	0.720 ± 0.02	0.087 ± 0.00	0.092 ± 0.01	***	ns	***	*
Ethyl tetradecanoate	0.006 ± 0.00	0.006 ± 0.00	0.005 ± 0.00	0.005 ± 0.00	0.000 ± 0.00	0.000 ± 0.00	0.006 ± 0.00	0.006 ± 0.00	0.006 ± 0.00	0.007 ± 0.00	0.000 ± 0.00	0.000 ± 0.00	***	ns	ns	ns
Ethyl vanillate	0.085 ± 0.00	0.084 ± 0.00	0.127 ± 0.00	0.123 ± 0.00	0.085 ± 0.00	0.088 ± 0.00	0.085 ± 0.00	0.090 ± 0.00	0.164 ± 0.00	0.195 ± 0.00	0.087 ± 0.00	0.088 ± 0.00	***	ns	***	ns
Ethyl dodecanoate	0.068 ± 0.01	0.116 ± 0.01	0.304 ± 0.02	0.487 ± 0.01	0.017 ± 0.00	0.017 ± 0.00	0.090 ± 0.01	0.112 ± 0.00	0.158 ± 0.01	0.097 ± 0.01	0.016 ± 0.00	0.016 ± 0.00	ns	***	**	ns
Ethyl hexadecanoate	0.010 ± 0.00	0.008 ± 0.00	0.008 ± 0.00	0.008 ± 0.00	0.000 ± 0.00	0.000 ± 0.00	0.009 ± 0.00	0.008 ± 0.00	0.011 ± 0.00	0.013 ± 0.00	0.000 ± 0.00	0.000 ± 0.00	***	***	***	ns
Phenethyl phenyl acetate	0.008 ± 0.00	0.001 ± 0.00	0.412 ± 0.00	0.282 ± 0.24	0.000 ± 0.00	0.000 ± 0.00	0.002 ± 0.00	0.001 ± 0.00	0.424 ± 0.00	0.492 ± 0.00	0.000 ± 0.00	0.000 ± 0.00	***	***	***	*
Ethyl cinnamate	0.000 ± 0.00	0.000 ± 0.00	0.000 ± 0.00	0.000 ± 0.00	0.000 ± 0.00	0.000 ± 0.00	0.000 ± 0.00	0.000 ± 0.00	0.000 ± 0.00	0.000 ± 0.00	0.892 ± 0.02	0.910 ± 0.07	***	ns	***	**
Ethyl acetate	4.694 ± 0.12	3.791 ± 0.08	4.348 ± 0.34	4.271 ± 0.18	4.412 ± 0.11	4.471 ± 0.08	4.726 ± 0.01	4.332 ± 0.02	4.424 ± 0.26	4.328 ± 0.23	4.590 ± 0.12	4.710 ± 0.07	***	ns	***	***
Ethyl lactate	0.922 ± 0.03	1.435 ± 0.09	0.459 ± 0.02	0.695 ± 0.04	1.352 ± 0.07	1.488 ± 0.07	1.500 ± 0.07	1.696 ± 0.09	1.293 ± 0.04	1.018 ± 0.05	3.206 ± 0.06	2.962 ± 0.09	***	***	**	ns
Diethyl succinate	0.142 ± 0.01	0.127 ± 0.01	0.116 ± 0.01	0.120 ± 0.01	0.292 ± 0.00	0.228 ± 0.01	0.174 ± 0.01	0.173 ± 0.01	0.155 ± 0.01	0.143 ± 0.01	0.393 ± 0.01	0.307 ± 0.02	***	ns	***	***
Crotonolactone	0.012 ± 0.00	0.006 ± 0.00	0.006 ± 0.00	0.006 ± 0.00	0.005 ± 0.00	0.006 ± 0.00	0.008 ± 0.00	0.007 ± 0.00	0.007 ± 0.00	0.009 ± 0.00	0.020 ± 0.00	0.022 ± 0.00	***	***	***	***
Butyrolactone	0.012 ± 0.00	0.004 ± 0.00	0.010 ± 0.00	0.002 ± 0.00	0.015 ± 0.00	0.015 ± 0.00	0.009 ± 0.00	0.014 ± 0.00	0.014 ± 0.00	0.012 ± 0.00	0.046 ± 0.00	0.052 ± 0.00	***	***	***	***
Nonalactone	1.133 ± 0.01	0.873 ± 0.07	1.088 ± 0.07	1.101 ± 0.13	0.940 ± 0.07	0.984 ± 0.09	1.186 ± 0.10	1.222 ± 0.04	1.310 ± 0.09	1.523 ± 0.08	0.901 ± 0.09	0.688 ± 0.04	***	*	***	ns
Decalactone	0.050 ± 0.00	0.052 ± 0.01	0.060 ± 0.01	0.048 ± 0.00	0.048 ± 0.00	0.064 ± 0.00	0.048 ± 0.00	0.061 ± 0.00	0.048 ± 0.00	0.049 ± 0.00	0.063 ± 0.00	0.083 ± 0.00	***	***	***	ns
*trans*-whisky lactone	0.067 ± 0.00	0.079 ± 0.00	0.110 ± 0.01	0.064 ± 0.00	0.122 ± 0.01	0.123 ± 0.01	0.057 ± 0.00	0.078 ± 0.00	0.149 ± 0.00	0.090 ± 0.00	0.285 ± 0.00	0.353 ± 0.02	**	ns	***	ns
*cis*-whisky lactone	0.630 ± 0.06	0.111 ± 0.02	5.648 ± 0.36	3.044 ± 0.07	1.439 ± 0.10	1.673 ± 0.04	1.204 ± 0.09	0.148 ± 0.02	7.431 ± 0.38	5.152 ± 0.17	3.895 ± 0.16	5.494 ± 0.19	***	*	***	ns
Guaiacol	0.374 ± 0.03	0.097 ± 0.01	0.138 ± 0.01	0.174 ± 0.03	0.747 ± 0.00	0.773 ± 0.00	0.358 ± 0.03	0.153 ± 0.01	0.300 ± 0.02	0.285 ± 0.02	1.080 ± 0.00	1.013 ± 0.00	***	ns	***	ns
2-methoxy-4-vinylphenol	1.881 ± 0.13	0.665 ± 0.06	0.383 ± 0.04	0.590 ± 0.03	1.354 ± 0.07	1.496 ± 0.10	1.453 ± 0.07	1.155 ± 0.07	1.174 ± 0.05	1.244 ± 0.05	2.066 ± 0.13	2.131 ± 0.13	***	ns	***	ns
Furfural	0.005 ± 0.00	0.002 ± 0.00	0.006 ± 0.00	0.006 ± 0.00	0.003 ± 0.00	0.005 ± 0.00	0.004 ± 0.00	0.004 ± 0.00	0.010 ± 0.00	0.011 ± 0.00	0.014 ± 0.00	0.017 ± 0.00	***	***	*	ns
5-methylfurfural	0.006 ± 0.00	0.010 ± 0.00	0.020 ± 0.00	0.044 ± 0.00	0.006 ± 0.00	0.014 ± 0.00	0.013 ± 0.00	0.021 ± 0.00	0.038 ± 0.00	0.065 ± 0.00	0.013 ± 0.00	0.030 ± 0.00	***	ns	***	*
5-HMF	0.000 ± 0.00	0.000 ± 0.00	0.000 ± 0.00	0.001 ± 0.00	0.000 ± 0.00	0.000 ± 0.00	0.001 ± 0.00	0.001 ± 0.00	0.000 ± 0.00	0.001 ± 0.00	0.001 ± 0.00	0.001 ± 0.00	***	**	***	***

Significant level: ns = non-significant; *** indicates *p* < 0.001; ** indicates *p* < 0.01; * indicates *p* < 0.05.

**Table 3:** Aroma series of wines aged with oak chips, staves, and barrels for 1.5 and 3 months. Multivariate analysis of variance (MANOVA) was conducted to study the influence of the type of ageing system (AS), the toast degree (TG), the ageing time (AT), and the interaction among factors (In) in the volatile aroma compound concentration. C: chips; S: staves; B: barrels; MT: medium toast; MPT: medium plus toast.

Aroma Series	CMT	CMPT	SMT	SMPT	BMT	BMPT	CMT	CMP	SMT	SMPT	BMT	BMPT	MANOVA			
	1.5 Months						3 Months						AS	TG	AT	In
Chemistry	17.90 ± 0.10	15.65 ± 0.15	15.97 ± 0.30	15.96 ± 0.38	18.26 ± 0.11	18.63 ± 0.29	18.05 ± 0.15	16.96 ± 0.11	17.45 ± 0.44	17.32 ± 0.27	20.20 ± 0.20	20.33 ± 0.26	***	***	***	***
Floral	6.91 ± 0.06	6.28 ± 0.22	6.98 ± 0.49	6.55 ± 0.31	9.26 ± 0.89	6.85 ± 0.36	7.01 ± 0.08	6.71 ± 0.13	7.72 ± 0.42	7.02 ± 0.18	9.32 ± 0.34	7.07 ± 0.37	***	***	*	ns
Green	1.88 ± 0.04	1.60 ± 0.00	1.59 ± 0.06	1.69 ± 0.05	0.97 ± 0.02	0.96 ± 0.06	1.84 ± 0.05	1.80 ± 0.05	1.92 ± 0.05	2.00 ± 0.07	1.32 ± 0.05	1.36 ± 0.05	***	ns	***	**
Caramelly	2.27 ± 0.12	0.78 ± 0.07	0.56 ± 0.05	0.82 ± 0.06	2.12 ± 0.07	2.30 ± 0.09	1.84 ± 0.05	1.34 ± 0.07	1.53 ± 0.04	1.61 ± 0.04	3.26 ± 0.12	3.27 ± 0.14	***	***	***	***
Fruity	106.72 ± 3.71	67.21 ± 3.98	97.21 ± 6.07	100.07 ± 4.28	77.02 ± 1.91	84.54 ± 0.69	113.98 ± 2.82	124.59 ± 6.14	146.80 ± 5.84	164.05 ± 3.55	96.81 ± 2.92	97.58 ± 5.42	***	ns	***	***
Creamy	2.60 ± 0.05	2.91 ± 0.14	2.05 ± 0.09	2.39 ± 0.17	3.04 ± 0.11	3.24 ± 0.06	3.54 ± 0.13	3.99 ± 0.09	3.42 ± 0.07	3.43 ± 0.15	5.55 ± 0.22	4.96 ± 0.10	***	**	***	***
Citrus	0.73 ± 0.02	1.12 ± 0.08	0.44 ± 0.04	0.90 ± 0.08	2.33 ± 0.14	2.68 ± 0.23	0.44 ± 0.04	0.89 ± 0.08	1.72 ± 2.32	1.00 ± 0.08	3.96 ± 0.36	7.30 ± 0.54	***	**	***	**
Honey	0.32 ± 0.02	0.40 ± 0.01	0.82 ± 0.02	0.70 ± 0.26	0.53 ± 0.03	0.54 ± 0.02	0.51 ± 0.01	0.48 ± 0.02	0.83 ± 0.01	0.80 ± 0.26	0.37 ± 0.00	0.54 ± 0.03	ns	ns	ns	ns
Waxy	9.70 ± 0.42	6.48 ± 0.34	9.23 ± 0.32	10.43 ± 0.45	2.00 ± 0.12	1.94 ± 0.09	13.59 ± 0.19	16.12 ± 0.21	16.58 ± 0.87	18.16 ± 0.47	2.25 ± 0.06	2.27 ± 0.07	***	*	***	***
Spicy	0.70 ± 0.05	0.19 ± 0.02	5.76 ± 0.36	3.11 ± 0.07	1.56 ± 0.11	1.80 ± 0.05	1.26 ± 0.09	0.23 ± 0.02	7.58 ± 0.39	5.24 ± 0.17	5.07 ± 0.17	6.76 ± 0.27	***	***	***	***
Woody	0.70 ± 0.05	0.19 ± 0.02	5.76 ± 0.36	3.11 ± 0.07	1.56 ± 0.11	1.80 ± 0.05	1.26 ± 0.09	0.23 ± 0.02	7.58 ± 0.39	5.24 ± 0.17	4.18 ± 0.16	5.85 ± 0.21	***	***	***	***

Significant level: ns = non-significant; *** indicates *p* < 0.001; ** indicates *p* < 0.01; * indicates *p* < 0.05.

## Data Availability

Data is contained within the article or supplementary material.

## Supplementary Materials

The following supporting information can be downloaded at https://www.mdpi.com/article/10.3390/foods13020196/s1, Table S1: Identification criteria and quantification.
