# Supplementary material for: Metabolomics of Red Wines Aged Traditionally, with Chips or Staves
Source: Foods. 2024 Jan 7;13(2):196. doi: 10.3390/foods13020196 (PMC10814756; doi:10.3390/foods13020196)
Supplement: Supplementary file 1 [file foods-13-00196-s001.zip › foods-2785784-supplementary.pdf]

Identification criteria and quantification

Aroma compounds were subjected to an identification process that involved three stages. Firstly, a tentative identification of metabolites was considered when the similarity values were equal or greater than 75% between the mass spectrum obtained for each chromatographic peak in samples and the mass spectral libraries (NIST-08 and Willey-7). Secondly, a difference lower than 15 units was considered between the linear retention index obtained for each compound through Van Den Dool & Kraft method (LRIc) and those reported (LRIr) in the NIST webbook of Chemistry. Finally, those compounds that did not meet these two requirements, were subjected to a definitive confirmation with the mass spectrum of their relative pure standards.

The quantification process was carried out through the internal standard quantification method, using calibration curves obtained with standard solutions subjected to the same analytical conditions as wine samples. The selection of target and qualifiers ions used for this purpose were performed with the Chemstation software (Agilent Technologies, Palo Alto, CA). The calibration curves from compounds with the most similar chemical structures and/or similar number of C atoms was used to estimate the concentration of those volatiles without pure compounds commercially available.

Supplementary Table S1. *Major and minor aroma compounds identified in the wines.*

	No.	Compound	Prv‡	CAS[c]	LRI[a]	LRI[b]	**Slope**	**Interception**	**R^2**	**LOD[d] (µg/L)**	**LOQ[e] (µg/L)**
***MAJOR COMPOUNDS**	**Alcohols**										
	1	Methanol	M	67-56-1	925	879	5.04E-03	6.0E-03	0.995	5.7E+03	19E+03
	2	Propan-1-ol	S	71-23-8	1068	1060	9.1E-03	5.0E-03	0.995	4.8E+03	16E+03
	3	2-methylpropan-1-ol (isobutanol)	S	78-83-1	1126	1108	9.5E-03	3.0E-03	0.999	1.8E+03	6.0E+03
	4	Isoamyl alcohols†	M	123-51-3	1243	1230	8.7E+03	9.0E-03	0.999	4.2E+03	14E+03
	5	2- Phenylethan-1-ol	S	60-12-8	2011	1892	11E-03	2E-03	0.999	3.6E+03	12E+03
	**Aldehydes and ketones**										
	6	Acetaldehyde	S	75-07-0	688	800	4.23E-03	-7.0E-04	0.999	7.8E+03	26E+03
	7	Acetoin	S	513-86-0	1337	1309	5.08E-03	-1E-03	0.997	9.1E+02	3E+03
	**Esters**										
	8	Ethyl acetate	S	141-78-6	906	885	4.96E-03	-3.0E-04	0.998	2.1e+03	7E+03
	9	Ethyl lactate	S	97-64-3	1354	1326	5.7E-03	3E-02	0.999	2.7E+03	9E+03
	10	Diethyl succinate	S	123-25-1	1730	1702	5.8E-03	2.8E-02	0.996	3.0E+03	10E+03
****MINOR COMPOUNDS**	**Alcohols**										
	11	E-2-hexen-1-ol	S	928-95-0	866.9	861	6.00E-04	2.60E-03	0.974	2	8
	12	E-3-hexen-1-ol	S	928-97-2	857	851	2.63E-03	-3.615E-03	0.968	0.54	1.8
	13	Hexan-1-ol	F	111-27-3	867	867	1.00E-03	0.00E+00	0.944	3	11
	14	Furfuryl alcohol	F	98-00-0	851	851	2.20E-02	8.50E-03	0.922	20	66
	15	Benzyl alcohol		100-51-6	1023	1020	1.883E-02	6.79E-04	0.942	1.08	3.6
	**Aldehydes and ketones**										
	16	Heptanal	S	111-71-7	903	901	7.90E-03	2.56E-03	0.961	0.4	1.3
	17	Benzaldehyde	S	100-52-7	958.9	959	5.10E-03	7.00E-03	0.988	3	10
	18	Octanal	S	124-13-0	1004	1004	9.10E-03	1.80E-02	0.961	0.2	0.6
	**Esters**										
	**ETHYL ESTERS**										
	19	Ethyl propanoate	S	105-37-3	708	705	2.20E-03	9.60E-03	0.967	12	39
	20	Ethyl butanoate	S	105-54-4	802	802	7.80E-03	1.03E-03	0.989	23	76
	21	Ethyl octanoate	F	106-32-1	1198	1196	1.78E-01	-4.70E-03	0.988	7	25
	22	Ethyl decanoate	F	110-38-3	1395	1397	1.69E-01	6.90E-03	0.976	0.5	1.7
	23	Ethyl dodecanoate	F	106-33-2	1594	1593	1.79E-01	7.10E-03	0.986	0.3	0.9
	24	Ethyl tetradecanoate	F	124-06-1	1793	1793	1.84E-01	6.51E-03	0.987	0.5	1.6
	25	Ethyl hexadecanoate	F	628-97-7	1992	1996	1.83E-01	-2.60E-03	0.971	0.6	2.1
	26	Ethyl vainillate[1]		617-05-00	2671	2676					

No.	Compound	Prv‡	CAS[c]	LRI[a]	LRI[b]	**Slope**	**Interception**	**R^2**	**LOD[d] (µg/L)**	**LOQ[e] (µg/L)**
27	Ethyl cinnamate		4610-69-9			7.78E-03	1.05E-02	0.989		
28	Ethyl isobutanoate	S	97-62-1	753	755	8.22E-02	1.64E-02	0.989	1	4
**HIGHER ALCOHOL ACETATES (HAAs)**										
29	Isobutyl acetate	S	110-19-0	772	781	4.80E-02	1.18E-02	0.984	0.5	1.6
30	2-Phenylethyl acetate	S	103-45-7	1256	1256	6.22E-02	4.10E-03	0.995	3	9
31	Phenethyl phenyl acetate	M	102-20-5	1919	1924	1.499E00	2.71E-02	0.983	0.54	1.8
**ISOAMYL ESTERS OF FATTY ACIDS (IEFAs)**										
32	3-Methylbutyl acetate (Isoamyl acetate)	F	123-92-2	875	876	4.80E-02	1.12E-02	0.984	22	75
**Lactones**										
33	5-H-furan-2-one (γ-crotonolactone)	S	497-23-4	913	916	1.00E-04	5.70E-03	0.972	9	29
34	Dihydrofuran-2(3H)-one (γ-butyrolactone)	F	96-48-0	918	922	2.00E-04	4.70E-04	0.974	14	47
35	5-Pentyloxolan-2-one (γ-nonalactone)	M	104-61-0	1363	1362	4.90E-03	-1.00E-04	0.943	1	4
36	γ-Decalactone	S	706-14-9	1470	1470	7.34E-02	-1.31E-02	0.983	1	4
37	E-whisky lactone	M	39638-67-0	1915	1914	1.50E-04	7.03E-03	0.970	1.62	5.4
38	Z-whisky lactone	M	55013-32-6	1290	1293	1.74E-04	6.81E-03	0.982	5.67	18.9
**Volatile phenols**										
39	2-Methoxyphenol *(Guaiacol)*	S	90-05-1	1091	1088	3.30E-03	1.50E-03	0.933	2	6
40	2-methoxy-4-vinylphenol	S	7786-61-0	1318	1315	1.81E-0.3	-3.14E-04	0.923	4.05	13.5
**Furanic compounds**										
41	Furan-2-carbaldehyde	F	98-01-1	831	830	2.40E-03	1.18E-02	0.978	22	72
42	5-Methylfurfural	M	620-02-0	967	964	6.38E-04	1.01E-02	0.942	95	315
43	5-(Hydroxymethyl)-2-furaldehyde	S	67-47-0	1226	1226	2.60E-03	1.28E-02	0.988	18	59

LRI: *Linear retention index* using definition of Van den Dool and Kratz (1963) in a *CPWAX57-CB capillary column (60 m/0.25 mm/0.40 µm. He) and **HP-5MS capillary column (30 m/0.25 mm/0.25 µm. He). .n.f. – not found in faced conditions. [a]Calculated values [b]Data collected from the NIST Webbook of Chemistry. http://webbook.nist.gov/chemistry. [c]CAS: Chemical Abstracts

Service number. [d] LOD: Limit of Detection. [e] LOQ: Limit of Quantification. † Isoamyl alcohols = 2-methylbutanol + 3-methylbutanol. [1]Quantified as ethyl decanoate.. ‡Prv: Standard providers. F: Fluka; M: Merck; S: Sigma-Aldrich.
